# Suppression of DLBCL Progression by the E3 Ligase Trim35 Is Mediated by CLOCK Degradation and NK Cell Infiltration

**DOI:** 10.1155/2021/9995869

**Published:** 2021-05-24

**Authors:** Xiyan Tan, Fuyang Cao, Feiyu Tang, Can Lu, Qiaoyan Yu, Songshan Feng, Zhanghuan Yang, Songming Chen, Xiang He, Jiang He, Liang Weng, Lunquan Sun

**Affiliations:** ^1^Xiangya Cancer Center, Xiangya Hospital, Central South University, Changsha 410008, China; ^2^Key Laboratory of Molecular Radiation Oncology Hunan Province, Changsha 410008, China; ^3^Department of Pathology, Xiangya Hospital, Central South University, Changsha 410078, China; ^4^Department of Neurosurgery, Xiangya Hospital, Central South University, Changsha 410078, China; ^5^Hunan International Science and Technology Collaboration Base of Precision Medicine for Cancer, Changsha 410008, China; ^6^Institute of Gerontological Cancer Research, National Clinical Research Center for Gerontology, Changsha 410008, China; ^7^Center for Molecular Imaging of Central South University, Xiangya Hospital, Changsha 410008, China

## Abstract

The majority of diffuse large B-cell lymphoma (DLBCL) patients develop relapsed or refractory disease after standard ruxolitinib, cyclophosphamide, doxorubicin, vincristine, and prednisone (R-CHOP) chemotherapy, which is partly related to a dysregulated tumor immune microenvironment. However, how the infiltration of immune cells is appropriately regulated is poorly understood. Herein, we show that the E3 ubiquitin ligase Trim35 is expressed at low levels in human DLBCL tissues. We also show that overexpression of Trim35 suppresses DLBCL cell proliferation and correlates with inferior survival in DLBCL patients. Our mechanistic study shows that Trim35 functions as an E3 ligase to mediate the ubiquitination and degradation of CLOCK, a key regulator of circadian rhythmicity. High expression of Trim35 correlates with NK cell infiltration in DLBCL, partly due to the degradation of CLOCK. Consistently, patients with high expression of CLOCK show poor overall survival. Overall, these findings suggest that Trim35 suppresses the progression of DLBCL by modulating the tumor immune microenvironment, indicating that it may be a promising diagnostic and prognostic biomarker in DLBCL.

## 1. Introduction

Diffuse large B-cell lymphoma (DLBCL), the most common subtype of non-Hodgkin lymphoma (NHL) in adults, has one of the highest mortality rates among cancers in the most developed areas of the world. Three gene expression subgroups, germinal center B cell-like (GCB-like), activated B cell-like (ABC-like), and primary mediastinal B cell lymphoma, have been identified, and the last two groups are also called non-GCB-like lymphoma [1, 2]. Patients with GCB-like DLBCL have significantly better overall survival than those with non-GCB-like DLBCL [3, 4]. Likewise, a clinical indicator of prognosis, the International Prognostic Indicator (IPI), has been successfully used to define prognostic subgroups of DLBCL. This indicator takes into account the patients' age and performance status, the extent and location of disease, and the serum LDH level [2]. Although current therapeutic strategies, including standard ruxolitinib, cyclophosphamide, doxorubicin, vincristine, and prednisone (R-CHOP) chemotherapy; unlabeled or radiolabeled monoclonal antibodies; and high-dose chemotherapy following autologous peripheral blood stem cell transplantation, have significantly improved the outcome of DLBCL, the majority of patients relapse or become resistant to prior therapies [5], which is partly due to the dysregulated tumor immune microenvironment. For example, the ABC-like DLBCL subtype was found to have inactivation of the CD58 gene, which indicates the loss of recognition of tumor cells by CTLs and NK cells and may be a cause of relapse or refractory disease in a patient with the ABC-like DLBCL subtype. Therefore, it is necessary to further investigate the underlying mechanism regulating the infiltration of immune cells into the tumor microenvironment.

Circadian rhythmicity is an approximately 24-hour cell-autonomous period driven by transcription-translation feedback loops of “circadian clock genes,” which are expressed in most cell types, including cells of the immune system [6–9]. The core circadian clock is an autoregulatory transcriptional feedback loop involving the activators CLOCK and BMAL1 and the repressor complex, including Per1, Per2, Cry1, and Cry2 [9–12]. The circadian system regulates virtually all physiological processes and influences the states of health and disease in humans. Recent advances have shown that circadian rhythmicity disruption contributes to disturbed immune responses, obesity, type 2 diabetes mellitus, and cancer. To date, how the expression of circadian clock genes is regulated in tumors is still poorly understood.

Ubiquitin is a small modifier molecule that labels proteins in a highly specific manner. Initially, ubiquitination was described as the process by which proteins are labeled for degradation by the proteasome [13], which is responsible for degrading 80-90% of intracellular proteins that are aberrantly folded or typically short-lived. Protein ubiquitination also regulates protein trafficking and protein-protein interactions via different linkages of polyubiquitin chains [14, 15]. The Ub-activation enzyme (E1), Ub-conjugation enzymes (E2), and Ub ligases (E3) are required to attach ubiquitin to a substrate [16, 17]. E3 ligases are considered to be the most important components of the ubiquitin conjugation machinery, as they bind directly to their target proteins and have substrate specificity. Protein ubiquitination affects many cellular processes, from gene transcription and DNA repair to the cell cycle and apoptosis [18]. Polyubiquitination often tags proteins for proteasomal degradation [19]. Recent advances indicate that the linear ubiquitin chain assembly complex (LUBAC) is crucially involved in B-cell lymphomagenesis through protection against DNA damage-induced cell death and is a suitable therapeutic target for B-cell lymphomas [20]. Moreover, recent research indicates that the CRL3-SPOP ubiquitin ligase complex suppresses the growth of diffuse large B-cells by negatively regulating MYD88/NF-*κ*B signaling [21]. Accumulating evidence has shown that ubiquitination is critical for DLBCL development. It has also been reported that the stability of the clock gene PER and CRY proteins are regulated by SCF E3 ubiquitin ligase complexes involving *β*-TrCP and FBXL3, respectively [22]. However, it remains largely unknown how the ubiquitination of circadian clock proteins is involved in DLBCL progression.

Tripartite motif 35 (Trim35), also called hemopoietic lineage switch (Hls5), is a member of the RING finger, B box, coiled coil (RBCC), or TRIM family E3 ligases expressed in a wide variety of hemopoietic cell types, including fetal liver progenitors [23]. Trim35 is reported to be a tumor suppressor gene [24]. Enforced expression of Trim35 in HeLa cells inhibits cell growth, clonogenicity, and tumorigenicity. Trim35 downregulation is a frequent event in hepatocellular carcinoma, and the expression level of Trim35 is negatively correlated with tumor size, histological grade, and serum alpha-fetoprotein concentration [24]. Moreover, TRIM family proteins are important effectors of innate immunity against viral infections. Recent findings revealed novel roles of Trim35; it catalyzes the Lys63- or Lys48-linked polyubiquitination of TRAF3, regulates RIG-I antiviral immunity, and is involved in the mechanism of defense against influenza A virus (IAV) infection [25]. However, the roles of Trim35 in the immune microenvironment and DLBCL have not been previously reported.

In this study, we identified that Trim35 is expressed at low levels in DLBCL tissues. Trim35 overexpression significantly inhibited DLBCL proliferation and is correlated with a better prognosis than Trim35 deficiency in DLBCL patients. In addition, we found that Trim35 interacts with CLOCK and promotes its ubiquitination and degradation. Furthermore, Trim35 and CLOCK promote and inhibit NK cell infiltration in DLBCL. Our study revealed how the ubiquitination of circadian clock proteins contributes to tumor immune microenvironment remodeling and DLBCL progression.

## 2. Methods

### 2.1. Patients

In total, 69 patients who were diagnosed with DLBCL at Xiangya Hospital of Central South University between 2013 and 2019 were included in this study. Clinical data were obtained from their medical records. The follow-up periods ranged from 4 to 88 months.

As an independent validation cohort, a total of 61 patients were diagnosed at DLBCL between 2013 and 2019 and, homogeneously treated with R-CHOP, were collected. The age of patients ranged from 10 to 86 years, and the follow-up duration was from 5 to 88 months.

### 2.2. Immunohistochemistry

Whole sections of representative formalin-fixed, paraffin-embedded (FFPE) tumor tissue blocks were submitted for immunohistochemistry (IHC). The immunohistochemical subgroup of DLBCL was determined to be non-GCB or GCB type according to Hans' criteria [26]. IHC was performed following standard protocols. In detail, immunohistochemical cytokeratin staining was performed on FFPE tissue using an in direct immunoperoxidase technique. Sections mounted on a slide were dewaxed in xylene, dehydrated in ethanol, boiled in 0.01 M citrate buffer (pH 6.0) for 30 minutes in a microwave oven, and then incubated with 3% hydrogen peroxide for 15 minutes. After washing with PBS, the slides were incubated in 5% normal BSA for 1 hour, followed by incubation overnight with rabbit polyclonal antibodies recognizing Trim35 (HPA019647, 1 : 200, Sigma-Aldrich, Shanghai, China), CLOCK (18094-1-AP,1 : 50, Protein-Tech, Wuhan, China), CD3 (Kit-0003, MaiXin Biotechnologies, Fuzhou, China), CD19 (MAB-0705, MaiXin Biotechnologies, Fuzhou, China), CD56 (MAB-0743, MaiXin Biotechnologies, Fuzhou, China), CD16 (16559-AP,1 : 100, Protein-Tech, Wuhan, China), and CD68 (Kit-0026, MaiXin Biotechnologies, Fuzhou, China). After washing, the sections were incubated with 3,3′-diaminobenzidine (DAB) (PV-6000D, ZSGB-BIO, Beijing, China). The sections were then counterstained with hematoxylin, dehydrated, cleared, and mounted. CD3 was used as a marker of T cells, CD19 was used as a marker of B-cells, CD56 and CD16 were used as markers of NK cells, and CD68 was used as a marker of tumor-associated macrophages (TAMs). Immunostaining for CD3, CD16, CD19, CD56, CD68, Trim35, and CLOCK was performed.

### 2.3. Cell Culture and Transfection

Pfeiffer cells (a human DLBCL cell line) were purchased from the American Type Culture Collection (ATCC) (Manassas, VA, USA). This cell line was cultured in RPMI 1640 medium (Gibco-Invitrogen), which was supplemented with penicillin (100 U/ml), streptomycin (100 *μ*g ml^−1^; Gibco-Invitrogen), and 10% fetal bovine serum (FBS); the cells were cultures in an incubator with 5% CO_2_ at 37°C. Human embryonic kidney 293FT cells were cultured in DMEM (Gibco-Invitrogen). All transfection experiments were performed using Lipofectamine 8000 (Beyotime Biotechnology, Shanghai, China) according to the manufacturer's recommendations. In detail, the cells were seeded in culture plates. When the cell density reached 70-80%, the cells were transfected with the plasmid. The cells were collected at 36-48 hours of posttransfection for western blot analyses.

For the generation of Pfeiffer cells stably expressing Flag tagged Trim35 or Trim35 KO cell lines, a lentiviral vector encoding Flag-Trim35 was transfected in a lentivirus packaging cell line, and the produced recombinant lentiviruses were then infected with the recombinant lentiviruses followed by a selection of infected cells using puromycin (A1113803, Thermo Fisher Scientific, Shanghai, China).

### 2.4. Plasmids and Small Guide RNAs (sgRNAs)

To overexpress Trim35, the coding regions were amplified from the cDNA of 293FT cells, and Trim35 was cloned into the PQCXIP-Flag vector between the SalI and BamHI sites. And the Trim35 sgRNA was constructed by annealing double complementary oligomers to encode sgRNA into the AgeI and EcoRI sites of the lentiCRISPR V2 vector, which is referred to as pSilencer throughout this report. sgRNAs were designed using the CRISPR tool (http://crispr.mit.edu) to minimize potential off-target effects. sgRNA sequences and genomic primers were shown as follows: Trim35 sgRNA (CACGTCGGGACTCCGCTCCA).

### 2.5. Cell Viability Assay

Cell viability was measured by using CCK-8 assays. Briefly, cell suspensions with a density of 4,000 cells per well were seeded in 96-well plates and incubated at 37°C. After overnight incubation, the cells were treated under the indicated conditions. At the end of the treatment, CCK-8 assays were used to calculate the number of viable cells by measuring the absorbance at 450 nm and normalized to the absorbance of a blank (fill with CCK-8 reagent only).

### 2.6. RNA Preparation and Quantitative Real-Time Polymerase Chain Reaction (PCR)

Total RNA was extracted from DLBCL cell lines by using TRIzol (Invitrogen) according to the manufacturer's instructions and was inversely transcribed to cDNA using PrimeScript RT-polymerase (RR047A-6, Takara, Dalian, China). The mRNA level of CLOCK was detected by using the iTaq™ Universal SYBR® Green (Bio-Rad), and GAPDH was used as an internal control.

The following primers were used for real-time quantitative reverse transcription PCR (qRT-PCR):

1. GAPDH primer:

- Forward: 5′- GGAGCGAGATCCCTCCAAAAT
- Reverse: 5′- GGCTGTTGTCATACTTCTCATGG

(2) CLOCK primer:

- Forward: 5′- TGCGAGGAACAATAGACCCAA
- Reverse: 5′- ATGGCCTATGTGTGCGTTGTA

### 2.7. Immunoblotting and Immunoprecipitation (IP)

For IP, extraction of proteins with a modified buffer from cultured cells was followed by IP and immunoblotting with the corresponding antibodies. Rabbit polyclonal antibodies recognizing Trim35 (HPA019647, 1 : 1000, Sigma-Aldrich, Shanghai, China) were obtained from Abcam; those for CLOCK (18094-1-AP,1 : 1000, Protein-Tech, Wuhan, China), Cryptochrome 2 (13997-1-AP,1 : 1000, Protein-Tech, Wuhan, China), and ARNTL (14268-AP,1 : 1000, Protein-Tech, Wuhan, China) were obtained from Protein Tech. Mouse polyclonal antibodies recognizing PER2 (67513-1-Ig,1 : 5000, Protein-Tech, Wuhan, China), GAPDH (60004-1-Ig,1 : 3000, Protein-Tech, Wuhan, China), and Flag (20543-1-AP, 1 : 3000, Protein-Tech, Wuhan, China) were purchased from Protein Tech.

### 2.8. In Vitro sgRNA Testing

In vitro testing of sgRNA was performed by lentivirus infection into the human DLBCL cell line Pfeiffer. Genomic DNA was harvested by DNA Extraction Kit (B518215, Sangon Biotech, Shanghai, China) and used as a PCR template, followed by T7 Endonuclease I (T7EI) assay.

### 2.9. T7 Endonuclease I (T7EI) Assay

PCR amplicons of target sites were purified and used as an input to the T7 Endonuclease I (T7EI) (D0508S, Genome-Editing Mutation Detection Kit, Beyotime Biotechnology, Shanghai, China). The manufacturer's recommended protocol was followed. T7 EI-treated products were run on a 2% agarose gel (Invitrogen), stained with nuclear acid dye, and imaged on a gel imager (Bio-Rad).

### 2.10. TIMER Analysis

The relationship between Trim35 expression and tumor-infiltrating immune cells (TIICs) in 32 cancer types was determined using the TIMER (https://cistrome.shinyapps.io/timer/) [27]. TIMER infers the abundance of TIICs applying the statistical analysis of gene expression profiles [28]. We analyzed the association between the level of Trim35 gene expression and the abundance of infiltrating immune cells, including tumor-associated macrophages (TAMs), monocytes, Tregs, myeloid dendritic cells (DCs), NK cells, B-cells, and CD4^+^ T cells based on the expression of specific marker genes in different cancers including DLBCL. The marker genes used for the analysis of the tumor-infiltrating immune cells including T cells, B-cells, monocytes, TAMs, M1 macrophages, M2 macrophages, natural killer (NK) cells, dendritic cells (DCs), Tregs, and myeloid-derived suppressor cells (MDSCs) were based on data from previous studies [29, 30].

### 2.11. Statistical Analysis

SPSS 24.0 was used to perform all statistical analyses. Numerical data are expressed as the mean ± standard error and were calculated using a two-tailed Student's **t**-test, chi-square test, and Spearman correlation analysis. **p** values are indicated by asterisks in the figures: ^∗^*p* < 0.05, ^∗∗^*p* < 0.01, and ^∗∗∗^*p* < 0.001. All experiments were repeated at least 3 times.

## 3. Results

### 3.1. Low Expression of Trim35 Is Correlated with a Poor Prognosis in DLBCL

**Figure 1:**
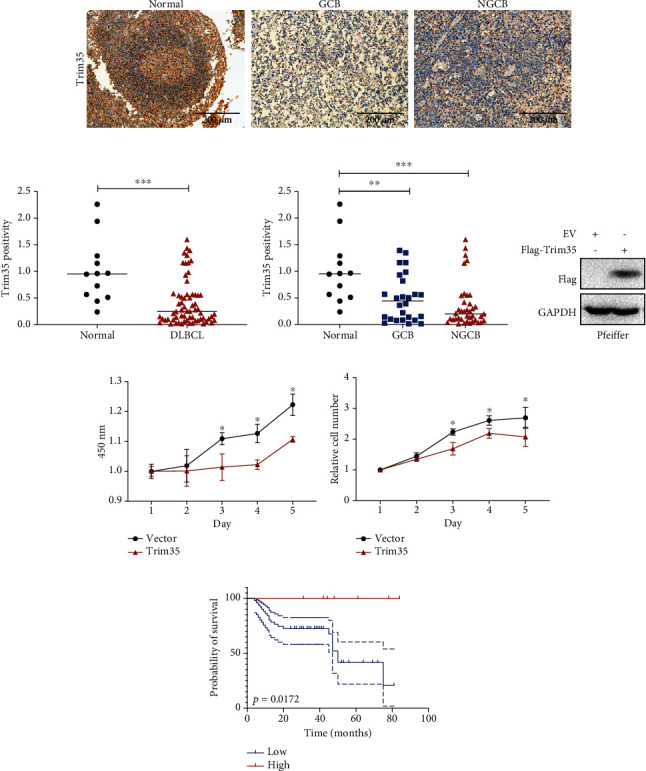
Low expression of Trim35 is correlated with poor prognosis in DLBCL. (a) Immunohistochemical staining for Trim35 in human DLBCL tissues and normal lymph nodes. The specific Trim35 signal is shown in brown (DAB staining), and counterstaining was performed using hematoxylin (blue nuclei). Scale bar, 200 *μ*m. (b, c) Summary for the entire cohort of DLBCL patients, as assessed by IHC. The *p* value was calculated by the chi-square test. (d) Pfeiffer cells were infected with virus expressing Flag-Trim35 or empty vector. After being selected, the protein extracts were immunoblotted for the indicated proteins. (e) CCK-8 assays of stable Trim35-overexpressing cells and empty vector-expressing cells. (f) Growth curves of stable Trim35 overexpression group and empty vector group were counted. (g) Overall survival analysis of patients with high (red) versus low (blue) Trim35 protein expression based on IHC staining. Samples with Trim35 staining in <5% of cells were considered to have low expression, and those with staining >5% were considered to have high expression. All experiments were repeated at least 3 times. ^NS^*p* < 0.05, ^∗^*p* < 0.05, ^∗∗^*p* < 0.01, and ^∗∗∗^*p* < 0.001.

To assess the effect of Trim35 on DLBCL cell growth, we established Pfeiffer cells stably expressing Trim35 (Trim35-OE) by lentiviral transduction (Figure 1(d)). Then, CCK-8 assays and cell counting analysis were performed to assess the effect of Trim35 on DLBCL cell proliferation. The results showed that overexpression of Trim35 greatly inhibited the proliferation of DLBCL cells (Figures 1(e) and 1(f)), which indicated that Trim35 may be involved in the suppression of DLBCL. To confirm the relevance of Trim35 to DLBCL, we subjected clinical tissue samples from 61 patients with primary DLBCL to IHC analysis. Compared with that in adjacent normal lymph nodes, nuclear Trim35 immunoreactivity in tumor tissues was markedly lower (Figures 1(a) and 1(b)). Notably, nongerminal center B-cell type (NGCB) DLBCL tissues expressed lower Trim35 levels than GCB DLBCL tissues (Figure 1(c)). Furthermore, Kaplan-Meier survival analysis demonstrated that high expression of Trim35 was significantly associated with a longer OS in DLBCL patients (*p* = 0.0172) (Figure 1(g)). Next, in a monofactor analysis, sex and cell of origin had no prognostic significance. However, cancer stage at diagnosis, serum LDH, and IPI score were found to be independent prognostic indicators for OS (cancer stage of diagnosis, *p* = 0.0132, HR = 3.530 [95% CI, 1.521-8.193]; serum LDH, *p* = 0.036, HR = 2.116 [95% CI, 0.8643-5.178]; and IPI score, *p* = 0.0078, HR = 2.906 [95% CI, 1.065-7.931]) (Table 1). Overall, these data showed Trim35 functions as a suppressor of DLBCL.

### 3.2. Trim35 Is Positively Associated with NK Cell Infiltration and Inhibits DLBCL Progression

**Figure 2:**
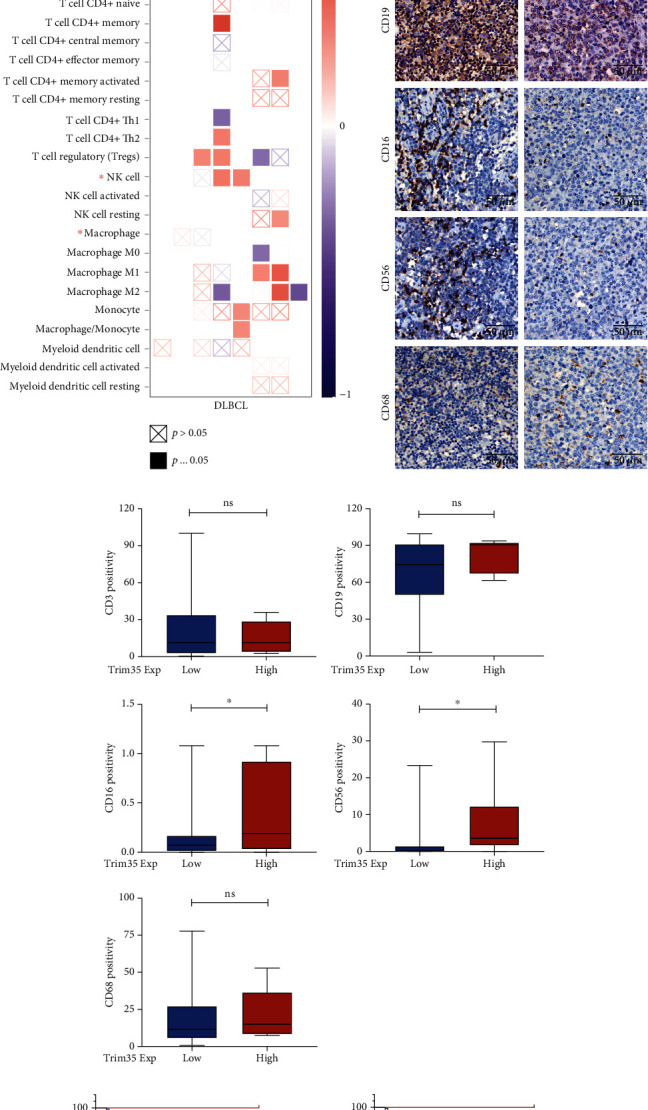
Trim35 is positively associated with NK cell infiltration and inhibits DLBCL progression. (a) Correlation of Trim35 expression with immune cells in DLBCL. (b) Representative IHC images of tumor-infiltrating CD3^+^, CD19^+^, CD16^+^, CD56^+^, and CD68^+^ cells from two patients with high and low expression of Trim35. Scale bar, 50 *μ*m. (c) The difference in CD3/CD19/CD16/CD56/CD68 positivity between the Trim35 subgroups was assessed by unpaired t test analysis. (d, e) Kaplan-Meier OS analysis in DLBCL with respect to phenotypic characteristics. Analysis of an NK cell marker (CD56^+^ and CD16^+^) was performed with evaluable immunostaining. ^NS^*p* < 0.05, ^∗^*p* < 0.05, ^∗∗^*p* < 0.01, and ^∗∗∗^*p* < 0.001.

To address how Trim35 suppresses DLBCL progression, we assessed whether Trim35 upregulation leads to antitumor effects by remodeling the immune microenvironment. To verify this hypothesis, we conducted Gene-Immune Analysis using the Tumor IMmune Estimation Resource (TIMER) (https://cistrome.shinyapps.io/timer/). As expected, the results showed that the expression of Trim35 was highly correlated with the infiltration of memory B-cells, CD4^+^ (nonregulatory) T cells, CD4^+^ memory T cells, resting CD4^+^ memory T cells, CD4^+^ Th2 cells, natural killer (NK) cells, resting NK cells, M1 macrophages, and monocytes (Figure 2(a)). To verify the correlation of Trim35 expression and immune cell infiltration, IHC was conducted to determine the numbers of tumor-infiltrating T cells (CD3^+^), B-cells (CD19^+^), macrophages (CD68^+^), and NK cells (CD16^+^ and CD56^+^) [31] in DLBCL. Representative IHC images are shown in Figure 2(b), and the cell densities (cell counts/mm) in 61 patients with DLBCL were quantified and plotted; the results showed a positive correlation between tumor-infiltrating NK cells (CD16^+^ and CD56^+^) and Trim35 (Figure 2(c)). Furthermore, better overall survival was observed in the patients with high infiltration of NK cells (CD56 positivity > 15%) than in those with low infiltration (CD56 positivity < 15%) (*p* = 0.0205). And the better overall survival was correlated with high expression of CD16 (samples with CD16 positivity < 0.8% of cells were considered to have low expression, and those with staining > 0.8% were considered to have high expression) (Figures 2(d) and 2(e)). Taken together, these results showed that Trim35 may suppress DLBCL progression by regulating NK cell infiltration.

### 3.3. Trim35 Is an E3 Ubiquitin Ligase for CLOCK and Promotes Its Proteasomal Degradation

**Figure 3:**
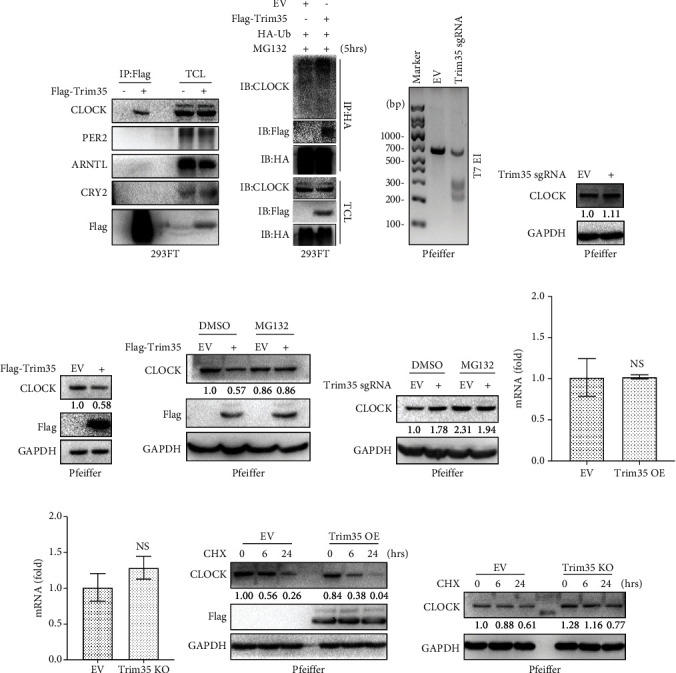
Trim35 controls the ubiquitination and degradation of CLOCK. (a) 293FT cells were transfected with Flag-tagged Trim35 or an empty vector. Forty-eight hours after transfection, cell lysates were prepared and subjected to IP with anti-Flag beads and immunoblotting as indicated. (b) Immunoblotting of lysed 293FT cells transfected with Trim35 or empty vector, along with HA-Ub. Cell lysates were prepared and subjected to IP with anti-HA beads and immunoblotting as indicated. (c) Trim35 sgRNAs are designed to target the human Trim35 loci. Agarose gel shows modification at both loci in transfected cells. (d) Pfeiffer cells were infected with viruses expressing Trim35 sgRNA or a control sgRNA and selected; cell lysates were immunoblotted for the indicated proteins. Densitometry quantified protein bands, presented as relative expression. (e) Pfeiffer cells were infected with Flag-Trim35 expression virus. Protein extracts were immunoblotted for the indicated proteins. CLOCK bands were quantified by densitometry and presented as relative expression. Pfeiffer cells with stable Trim35 overexpression (f) and Trim35-depleted Pfeiffer cells (g) were treated with a proteasome inhibitor MG132 (10 *μ*g/mL) during the last 5 hours before lysis. CLOCK protein levels were detected and analyzed. Protein bands were quantified by densitometry and presented as relative expression. (h, i) The same cells as described in (e, d) were used for qRT-PCR analysis of CLOCK mRNA expression. Results are normalized to GAPDH mRNA level and expressed fold changes in mRNA expression compared with control. (j, k) The same cells as described in (e, d) were cultured for 6 h and 24 h before being further incubated with cycloheximide (CHX) for the indicated time points. The level of CLOCK at different time points was detected by western blot. Densitometry quantified CLOCK bands, presented as relative expression. Each experiment was successfully carried out three times. ^NS^*p* > 0.05, ^∗^*p* < 0.05, ^∗∗^*p* < 0.01, and ^∗∗∗^*p* < 0.001.

The clock genes are reported to be involved in the regulation of the tumor microenvironment and cancer progression. To explore molecular mediators of the tumor suppressive function of Trim35, we overexpressed Flag-Trim35 in 293FT cells and then performed IP analysis to check the interaction between Trim35 and several clock proteins (Figure 3(a)). Among these proteins, CLOCK was successfully coimmunoprecipitated by Flag-Trim35, suggesting an interaction between the two proteins. Trim35 is reported as an E3 ligase, so we explored whether Trim35 could promote the ubiquitination and degradation of CLOCK. Trim35 overexpression induced CLOCK ubiquitination (Figure 3(b)) in 293FT cells. Besides, Trim35 knockout cell line was constructed, to verify the effective sgRNA for Trim35; we quantified the editing efficiency using the T7 Endonuclease I assay. The Trim35 sgRNAs were capable of inducing indels in specific targeted loci (Figure 3(c)). Consistent with this finding, depletion of Trim35 increased the CLOCK protein level in DLBCL cell lines (Figure 3(d)). Interestingly, stable overexpression of Trim35 in DLBCL cell lines reduced the endogenous protein level of CLOCK (Figure 3(e)). These observations suggested that Trim35 is involved in regulating CLOCK expression. Next, we analyzed endogenous CLOCK ubiquitination in the DLBCL cell line in the presence or absence of the proteasome inhibitor MG132. As expected, we found that Trim35-induced suppression of CLOCK could be efficiently abolished by MG132 (Figures 3(f) and 3(g)), which suggested that CLOCK was degraded through proteasome-mediated proteolysis degradation pathway. Further, we observed Trim35 has no effect on the mRNA level of CLOCK (Figures 3(h) and 3(i)). To further define whether Trim35 modulated the stability of endogenous CLOCK, we inhibited *de novo* protein levels of CLOCK in the Trim35 overexpressed and Trim35 knock out cells. Our data showed that Trim35 significantly induced degradation of CLOCK proteins in these cycloheximide-treated DLBCL cells (Figures 3(j) and 3(k)). These results suggest that Trim35 functions as an E3 ligase to promote the degradation of CLOCK through the proteasome pathway.

### 3.4. CLOCK Is Highly Expressed in DLBCL and Is Negatively Correlated with NK Cell Infiltration

**Figure 4:**
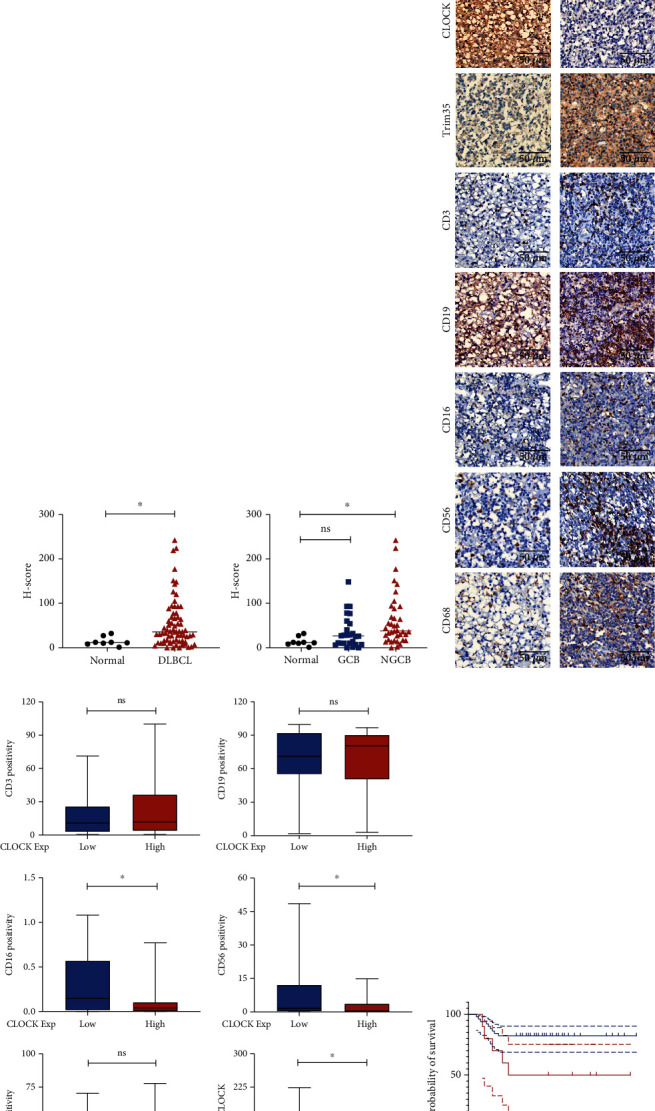
CLOCK is highly expressed in DLBCL and negatively correlated with NK cell infiltration. (a) Immunohistochemical staining for CLOCK in human DLBCL tissues and normal lymph node tissues. The specific CLOCK signal is seen in brown (DAB staining), and counterstaining was performed using hematoxylin (blue nuclei). Scale bar, 200 *μ*m. (b) Summary for the entire cohort of DLBCL patients, as assessed by IHC. The *p* value was calculated by the chi-square test. (c) Representative IHC images of Trim35 and tumor-infiltrating CD3^+^, CD19^+^, CD16^+^, CD56^+^, and CD68^+^ cells from two patients with high and low expression of CLOCK. Scale bar, 50 *μ*m. (d) Differences in CD3/CD19/CD16/CD56/CD68 positivity between the two CLOCK subgroups were assessed by an unpaired *t* test. (e) Overall survival of patients with high (red) versus low (blue) CLOCK expression based on IHC staining. ^NS^*p* > 0.05, ^∗^*p* < 0.05, ^∗∗^*p* < 0.01, and ^∗∗∗^*p* < 0.001.

To investigate whether Trim35 suppresses DLBCL progression via CLOCK, we first assessed the clinical relevance of trim35 in human DLBCL. CLOCK expression was analyzed in human DLBCL samples, and representative IHC images are shown in Figure 4(a). The expression of CLOCK was significantly increased in DLBCL tissues compared with normal lymph node tissue (Figure 4(b)) and was inversely correlated with the expression of Trim35. Next, to further clarify the relevance of CLOCK in the immune microenvironment of human DLBCL, we determined the correlation between CLOCK expression and markers of several immune cells. Consistently, we found a negative correlation between CLOCK and CD56 and CD16, two biomarkers of NK cells (Figure 4(d)), and the representative IHC image is shown in (Figure 4(c)). Otherwise, the overall survival of patients with high CLOCK expression was significantly worse than that of patients with low CLOCK expression (*p* = 0.0309) (Figure 4(e)). These results indicate that Trim35 may remodel the tumor immune microenvironment via CLOCK.

## 4. Discussion

Our present study found a significant decrease in Trim35 expression in human DLBCL tissues compared with normal lymph node tissues, indicating the clinical relevance of Trim35 in human lymphomagenesis. We further showed that overexpression of Trim35 in human DLBCL cells can suppress their proliferation and correlates with poor survival in DLBCL. A mechanistic study showed that Trim35 functions as an E3 ligase to mediate the ubiquitination and degradation of CLOCK. Consistently, bioinformatics methods and IHC analysis of human DLBCL samples revealed that Trim35 and CLOCK expression were positively and negatively correlated with NK cell infiltration, respectively. In addition, our study confirmed that Trim35 and CLOCK expression and NK cell infiltration are independent prognostic variables of DLBCL. Future studies are required to uncover the detailed mechanism by which CLOCK regulates NK cell infiltration in DLBCL.

Human tumors can escape immune surveillance to enhance their survival [32, 33]. Anticancer immunotherapy has been shown to be a powerful way to cure cancer. NK cells reside in the peripheral blood and in some lymphoid and nonlymphoid organs and are promptly recruited to tumor sites as an important component of tumor immunosurveillance [31, 34]. Activated NK cells also rapidly secrete a variety of cytokines and chemokines, such as interferon *γ* (IFN*γ*), that promote the recruitment and activation of other participants in the antitumor response [35]. Their effector functions also represent a crucial factor in determining the response to anticancer therapy; for example, NK cells play an important role in rituximab-dependent killing of lymphoma cells via an antibody-dependent cellular cytotoxicity (ADCC) mechanism [36, 37]. A previous study also confirmed that an absolute decrease in NK cell count was predictive of no response and of shorter event-free survival and progression-free survival (PFS) in DLBCL, which suggested that the peripheral blood natural killer cell count (NKCC) could be a valuable biomarker in clinical practice and could pave the way for the development of novel combination treatment approaches for B-cell non-Hodgkin lymphoma (B-NHL) that are aimed at enhancing the activity of anti-CD20 antibodies by providing them with a sufficient number of functional effector cells [38–40]. Furthermore, the prognostic and therapeutic significance of the host response represented by NK cell infiltration has been described in Hodgkin lymphoma and DLBCL [41]. All of these findings highlight the crucial role of NK cells in DLBCL.

The target protein CLOCK is a key regulator of circadian rhythm, which is a biological mechanism that dictates an array of rhythmic physiological processes. The feature of this circadian control relies on cellular metabolism, both within the tumor microenvironment and organism systemically [42]. Previous studies have characterized the impact of the circadian clock on tumorigenesis and specific immune cells. For example, a recent study revealed that CD4^+^ and CD8^+^ T cells are correlated with core clock molecules, especially in lung adenocarcinomas and lung squamous cell carcinomas [43]. Additionally, it was reported that the tumor growth rate increased and latency decreased under circadian disruption conditions compared to normal light-dark (LD) schedules in a murine melanoma model [44]. However, little is known about the role of the clock gene in tumor microenvironment regulation and DLBCL progression. Our results reveal that the circadian gene CLOCK has a strong inverse correlation with NK cell infiltration in DLBCL tissues. In addition, we found that CLOCK is degraded by Trim35 via the ubiquitination-proteasome pathway. Consistently, CLOCK is overexpressed in DLBCL patients and is inversely correlated with the expression of Trim35.

To our knowledge, Trim35 is the first E3 ligase of CLOCK identified so far, which also extends our understanding of the suppressive role of Trim35 in tumor progression.

## 5. Conclusions

Overall, the present study revealed that Trim35 functions as an E3 ubiquitin ligase of CLOCK to promote its ubiquitination and proteasomal degradation; this suppresses DLBCL development by enhancing the infiltration of cytotoxic NK cells into tumors. Trim35 is a promising prognostic biomarker in DLBCL, and future studies should be conducted to further uncover the detailed mechanism by which the ubiquitination of clock proteins is related to remodeling of the tumor microenvironment and treatment response.

## Figures and Tables

**Table 1:** Monofactor analysis of overall (OS) in patients with DLBCL.

Characteristics	Variables	Total (*n* = 61)	Hazard ratio (95% CI)	*p* value
Age (years)	<60	46	1.429 (0.5667-3.604)	0.4046
	>60	15		
Sex	Male	39	0.9404 (0.3972-2.226)	0.8881
	Female	22		
Cell of origin	GCB	24	0.5581 (0.2379-1.309)	0.1587
	Non-GCB	37		
Cancer stage at diagnosis	I/II	23	3.530 (1.521-8.193)	0.0132
	III/IV	38		
Serum LDH	Normal	37	2.116 (0.8643-5.178)	0.036
	Elevated	24		
IPI score	0/1/2	42	2.906 (1.065-7.931)	0.0078
	3/4/5	19		

## Data Availability

The data used to support the finding of this study are available from the corresponding author upon request.
